# Productivity and Phytochemicals of *Asclepias curassavica* in Response to Compost and Silver Nanoparticles Application: HPLC Analysis and Antibacterial Activity of Extracts

**DOI:** 10.3390/plants12122274

**Published:** 2023-06-11

**Authors:** Mervat El-Hefny, Abeer A. Mohamed, Ahmed Abdelkhalek, Mohamed Z. M. Salem

**Affiliations:** 1 Department of Floriculture, Ornamental Horticulture and Garden Design, Faculty of Agriculture (El-Shatby), Alexandria University, Alexandria 21545, Egypt; mervat.mohamed@alexu.edu.eg; 2 Plant Pathology Institute, Agricultural Research Center (ARC), Alexandria 21616, Egypt; abeer_pcr@yahoo.com; 3 Plant Protection and Biomolecular Diagnosis Department, ALCRI, City of Scientific Research and Technological Applications, New Borg El Arab City 21934, Egypt; aabdelkhalek@srtacity.sci.eg; 4 Forestry and Wood Technology Department, Faculty of Agriculture (El-Shatby), Alexandria University, Alexandria 21545, Egypt

**Keywords:** *Asclepias curassavica*, nanosilver, compost, methanolic extract, antibacterial activity, phenolic, flavonoids

## Abstract

The application of compost and metallic nanoparticles has a significant impact on the productivity and chemical composition of horticulture plants. In two subsequent growing seasons, 2020 and 2021, the productivity of *Asclepias curassavica* L. plants treated with various concentrations of silver nanoparticles (AgNPs) and compost was assessed. In the pot experiments, the soil was amended with 25% or 50% compost, and the plants were sprayed with 10, 20, and 30 mg/L of AgNPs. Scanning electron microscopy (SEM), transmission electron microscopy (TEM), X-ray diffraction analysis (XRD), and dynamic light scattering (DLS) were used to characterize AgNPs. The TEM measurements of AgNPs showed that the particles had spherical forms and ranged in size from roughly 5 to 16 nm. Leaf methanol extracts (LMEs) were prepared from the treated plants and assayed against the growth of two soft rot bacteria, *Dickeya solani* and *Pectobacterium atrosepticum*. The maximum plant height, diameter, number of branches/plant, total fresh weight (g), total dry weight (g), and leaf area (cm^2^) was recorded when levels of 25% compost + AgNPs 20 mg/L, 25% compost, or 50% + AgNPs 20 mg/L, 25% compost + AgNPs 30 mg/L or 50% compost + AgNPs 20 mg/L, 50% compost + AgNPs 20 mg/L, 50% compost + AgNPs 30 or 20 mg/L, and 25% compost + AgNPs 30 mg/L, respectively, were applied. The plants treated with 25% or 50% compost + 30 mg/L AgNPs showed a high chlorophyll content, while the plants treated with 50% compost + AgNPs 30 mg/L or 20 mg/L showed the highest extract percentages. The highest inhibition zones (IZs), 2.43 and 2.2 cm, against the growth of *D. solani* were observed in the LMEs (4000 mg/L) extracted from the plants treated with compost (*v*/*v*) + AgNPs (mg/L) at the levels of 50% + 30 and 25% + 30, respectively. The highest IZs, 2.76 and 2.73 cm, against the growth of *P. atrosepticum* were observed in the LMEs (4000 mg/L) extracted from the plants treated at the levels of 50% + 30 and 25% + 30, respectively. Several phenolic compounds such as syringic acid, p-coumaric acid, chlorogenic acid, cinnamic acid, ellagic acid, caffeic acid, benzoic acid, gallic acid, ferulic acid, salicylic acid, pyrogallol, and catechol, as well as flavonoid compounds such as 7-hydroxyflavone, naringin, rutin, apigenin, quercetin, kaempferol, luteolin, hesperidin, catechin, and chrysoeriol, were identified in the LMEs as analyzed by HPLC with different concentrations according to the treatment of compost + AgNPs used for the plants. In conclusion, the specific criteria that were utilized to measure the growth of *A. curassavica* revealed the novelty of compost and AgNPs combination treatments, particularly at a concentration of 50% compost + AgNPs 30 mg/L or 20 mg/L, which is better for the growth and phytochemical production of *A. curassavica* in the field.

## 1. Introduction

*Asclepias curassavica* L. (Tropical milkweed), “Silky Gold” (Golden Butterfly weed), is an erect, evergreen subshrub belonging to the subfamily Asclepiadoideae, family Apocynaceae [1]. *A. curassavica* can reach a height of 1 m. Plants in the Apocynaceae family are generally the source of cardiac glycosides and have valuable therapeutic components. The isolation of several glycosides, including cardiac glycosides, phenols, saponins, steroids, tannins, terpenoids, and protein/amino acids, has been reported [2,3,4].

In South America, the plant’s root extracts are widely used as emetics and laxatives. When used for abdominal tumors and hemorrhages, milkweed is anti-ovulatory, astringent, and cardiotonic. The plant also contains a highly esterified polyhydroxypregnane glycoside that has antitumor and anticancer characteristics. It is used to treat lung diseases, bruises, wounds, skin ulcers, chronic cough, and headaches as well as diarrhea, dysentery, and chronic rheumatism [2,5].

The application of nanotechnology could increase agricultural productivity by improving the management of plant and animal production. The most researched and used nanoparticles for biosystems are silver nanoparticles (AgNPs) [6]. Compared to bulk silver, AgNPs have a high surface area and high antimicrobial activity [7]. Additionally, AgNPs’ antioxidant, antibacterial, antifungal, and antiviral activities play a part in agricultural crop protection [8,9,10,11]. They have been linked to improved harvests and increased crop yield in agriculture by regulating optimum nutrition for plants and promoting seed germination and plant growth [12,13,14,15,16].

When *Trigonella foenum-graecum* seeds were exposed to AgNPs at a concentration of 10 μg/mL, maximum seed germination, speed of germination, root length, root fresh weight, and root dry weight were observed [17,18]. A foliar spray of AgNPs at a concentration of 50 ppm in cowpea caused growth promotion and increased root nodulation [19]. This concentration of AgNPs also has a positive effect on fresh weight, shoot and root length, and the strength index of *Brassica juncea* seedlings [18].

Compost is commonly used as a soil amendment in horticultural and agricultural practices, and its effects on soil quality and plant growth include improved nutrient availability and uptake, increased competitiveness, decreased weed emergence, and reduced levels of heavy metals that are available to plants [20,21,22,23]. Compost is typically used as an efficient way to change soil properties, particularly to increase water-holding capacity and soil organic matter, improve soil structure, and increase infiltration and permeability [24,25,26,27,28].

Essential oils and natural extracts derived from plants have been used to control the growth of bacterial pathogens in potatoes (*Solanum tuberosum* L.) [11,29]. *Pectobacterium* and *Dickeya* cause soft rot diseases in potatoes and other horticultural crops [30]. *Pectobacterium* and *Dickeya solani* cause soft rot in tubers and top wilt in growing potato plants. As the soft rot spreads from the infected tuber to the plant through the vascular system, the wilt may occur quickly. Wilting may occur in some varieties even when there is no visible blackleg. A lower inoculum level as an infection threshold is thought to make *D. solani* more powerful than the *Pectobacterium* species [31,32]. The milkweed plant secretes thick, white, or milky latex that is rich in bioactive phytochemicals such as flavonoids, glycosides, simple phenols, tannins, and other substances [33]. The ethanolic extracts from *A. curassavica* produced the maximum mortality of the colonies within 7 days against the leaf-cutting ant *Atta sexdens rubropilosa* among extracts from *A. curassavica*, *Rosmarinus officinalis*, and *Equisetum* spp. [34]. As determined by DPPH, nitric oxide, and superoxide anion radicals, the hydroalcoholic extract of the aerial component of *A. curassavica* exhibited scavenging antioxidant activities [35]. *Trichomonas vaginalis* was resistant to the ethanol extract from the aerial parts of *A. curassavica*; however, it was effective against pain [36]. Using GC-MS, glycerol-3TMS ether, myristamide, L-(−)-arabitol, pentakis(3TMS) ether, D-(-)-fructopyranose, pentakis(3TMS), β-D-glucopyranose, TMS, myoinositol-TMS, glucosamine per-TMS, oleamide, N-3TMS, aucubin, hexakis(3TMS) ether, D-(+)-turanose, octakis(3TMS) ether, and sucrose, octakis(3TMS) ether was identified in the silylated alcoholic extract of *A. curassavica* leaves [37]. Cardenolides, which were isolated from *A. curassavica*, and their derivatives covenosigenin, glucopyranoside, and acospectoside play a significant role as termite antifeedants [38,39]. The extract included total polyphenols and flavonoids presenting 58.75 µg/mL and 150.1 µg/mL, respectively, and showed promise in the control of *Spodoptera frugiperda* J.E.Smith [40].

To the best of our knowledge, the *Asclepias curassavica* plant has not received much attention from researchers despite the fact that it has a variety of purposes in both ornamental gardening and medical bioactivity. AgNPs and compost are being applied to this plant for the first time.

Therefore, the aim of this experiment was to investigate the effect of compost and silver nanoparticles at various concentrations on *Asclepias curassavica* growth, and the phytochemical as well as biological activity of these substances against *Dickeya solani* and *Pectobacterium atrosepticum*, two bacteria that cause soft rot.

## 2. Results

### 2.1. UV-Vis and X-ray Diffraction (XRD) Analyses

**Figure 1:**
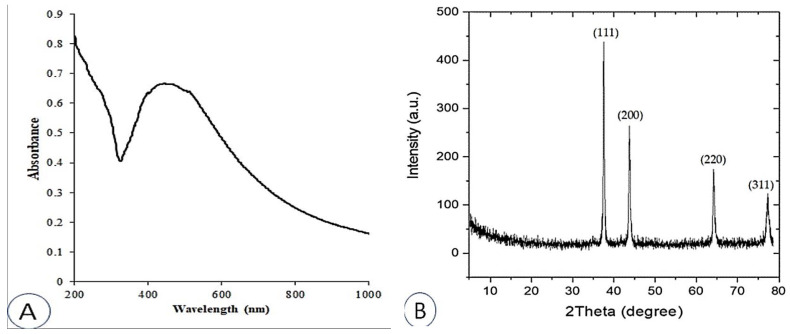
UV-VIS spectral analysis (**A**); X-ray diffraction (XRD) spectrum (**B**) of synthesized AgNPs.

The UV-Vis absorption spectra of the produced AgNPs were examined between 200 and 1000 nm (Figure 1A). The majority of AgNPs contain a surface plasmon resonance (SPR) band between 420 and 550 nm [41,42,43], which is caused by the excitation of free electrons. AgNPs were found to have an SPR value of 446 nm, which is consistent with results from several other studies [44,45,46].

The crystal structure and phase of nanoparticles, including AgNPs, are typically examined using XRD [47]. Figure 1B shows the XRD pattern of the synthesized AgNPs. The primary peaks, which correspond to the 111, 200, 220, and 311 planes, are located at (2θ) 37.51°, 42.81°, 66.44°, and 79.29°, respectively. These peaks confirm the formation of AgNPs.

The results are in agreement and demonstrate that most AgNPs have a face-centered cubic material (fcc) structure and are consistent with JCPDS card number 89 3722 [48]. The XRD properties are detailed in Table 1. Information collected from XRD measurements, including the prominent peak, the d-spacing, and the predicted 2θ value for AgNPs, are presented in Table 2. The findings, taken as a whole, provide evidence that the naturally occurring structure of the synthesized AgNPs is crystalline.

### 2.2. Particle Size Distribution and Morphological Characterization of the Synthesized AgNPs

**Figure 2:**
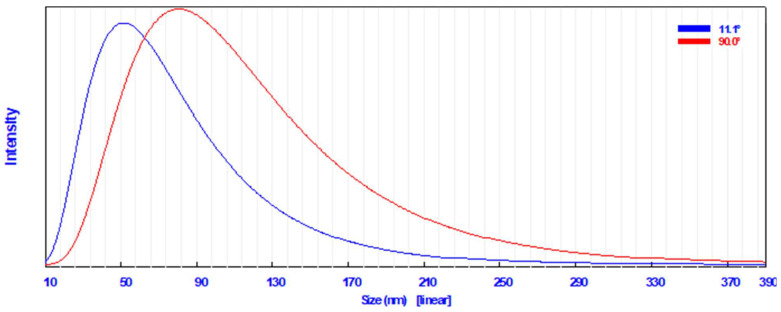
Particle size distribution using dynamic light scattering technique of the synthesized AgNPs.

A common technique for determining how the sizes of particles are dispersed in a colloidal fluid is dynamic light scattering (DLS) [49]. The DLS analysis in the current study revealed that the particle size distribution of the biosynthesized AgNPs in the aqueous medium was broad; however, there was a single peak distribution corresponding to the average particle size, which was found to be 57.3 nm at 11.1° (Figure 2). AgNPs had an average diameter of 87.6 nm at 90.0°.

**Figure 3:**
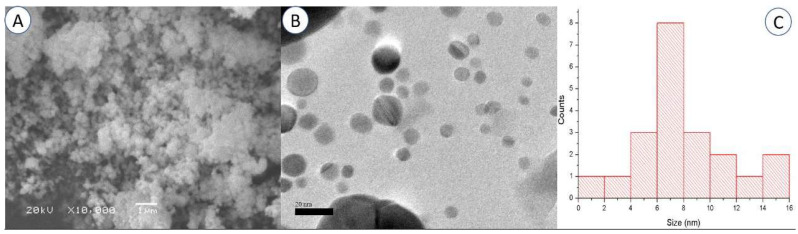
Scanning electron microscopy (**A**) and transmission electron microscopy (**B**) images of the synthesized AgNPs (Bar = 1 µm for SEM and 20 nm for TEM). Particle size distribution graph using TEM analysis (**C**).

One of the most effective methods for determining the appearance of the surface is scanning electron microscopy (SEM), which allows us to directly observe the nanoparticles [50]. The SEM investigation revealed that the powders with a lower degree of miscibility are composed of spherical-appearing pure AgNPs (Figure 3A). The SEM images demonstrated how the AgNPs combined to form lager particles. This effect could be explained by the existence of free static charges on the surface of the AgNPs [47,51].

According to the TEM findings, spherical AgNPs with a size range of roughly 5 to 16 nm were formed (Figure 3B). The particle size distribution graph using TEM analysis is presented in Figure 3C. The majority of the AgNPs were found to be spread out, and only a few were found to be clumped together in groups of different sizes. The difference in size between the DLS and TEM methods is acceptable because, unlike in TEM [52,53], the diameter of the particles in DLS is determined with a layer of solvent in the scattered phase.

### 2.3. FTIR Analysis

**Figure 4:**
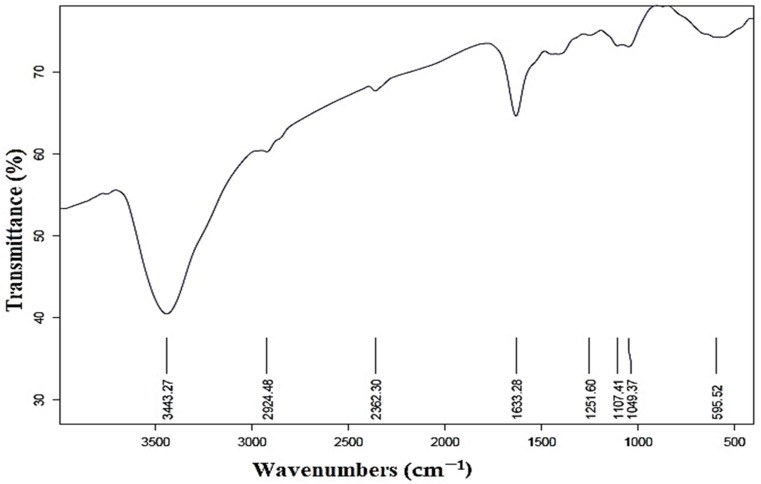
Fourier transform infrared spectra of prepared AgNPs.

As illustrated in Figure 4, the FTIR spectrum reveals numerous functional groups in various locations. The peaks in the 3443 cm^−1^ and 2924 cm^−1^ were attributed to the aldehyde-C-H- and O-H-stretching of alcohol compounds [54,55]. The existence of C=O groups (aldehydes and ketones) in the produced AgNPs was confirmed by the peak at 2362 cm^−1^ [40]. Furthermore, the peak found at 1633 cm^−1^ demonstrated the carbonyl group’s (C=O) stretching vibration and the amine group’s N-H bending [55]. The spectral bands observed at the 1107 and 1049 wavenumbers were assigned to the stretching vibrations of the C-O bond, as reported in previous studies [56,57]. Furthermore, it is plausible that the peak observed at 595 cm^−1^ may be attributed to the stretching of C-N bonds in amine functional groups, as suggested by previous research [58]. This investigation demonstrates the contradictory behavior of molecules that may be involved in the stabilization and reduction of silver nanoparticles [59].

### 2.4. Effect of Compost and AgNPs on Vegetative Growth of A. curassavica

According to the findings in Table 3, compost and AgNPs have a positive impact on the vegetative parameters of *A. curassavica* plants including plant height (cm), plant diameter (cm), branch number/plant, leaf fresh weight (g), leaf dry weight (g), and leaf area (cm^2^), which gradually increased as the concentration increased in comparison to untreated plants. In contrast, the higher plant height was obtained by treating plants with 50% compost + AgNPs 20 mg/L (58.05 cm) in the first season and 25% compost + AgNPs 20 mg/L in the second season (61.66 cm), while the lower plant height was obtained by treating plants with 0% compost + 10 mg/L AgNPs in both seasons (36.61 and 41.89 cm, respectively). In the first and second seasons, respectively, the application of the 50% compost + AgNPs 20 and 50% compost +30 mg/L treatments revealed the highest diameter values of *A. curassavica* plants with 43.69 cm and 45 cm, respectively.

The plants that were treated with 25% compost + AgNPs 30 mg/L in the first season and 50% compost + AgNPs 20 mg/L in the second season produced the highest number of branches/plant. The highest total fresh weight (g) was produced by the application of 50% compost + AgNPs 20 mg/L (62.73 g) followed by 50% compost + AgNPs 10 mg/L (59.97 g) in the first season, and 50% compost + AgNPs 30 mg/L (63.67 g) followed by 50% compost + AgNPs 20 mg/L (62.81 g) in the second season.

In both seasons, 50% compost + AgNPs 30 or 20 mg/L produced the greatest results for the total dry weight (g). In the first season, this result was 19.51 and 19.43 g, respectively, but in the second season, it was 24.10 and 22.91 g. Additionally, 25% compost + AgNPs 30 mg/L (16.2 and 16.2 cm^2^) had the greatest impact on leaf area (cm^2^) compared to the control (7.26 and 8.45 cm^2^) for both seasons.

### 2.5. Effect of AgNPs on Biochemical Constituent of A. curassavica

Results in Table 4 show that the compost and AgNPs significantly affected the chemical components of *A. curassavica* plants, including chlorophyll content (measured in SPAD units) and leaf methanol extract (LME%) (Table 4). The plants treated with 25% or 50% compost + 30 mg/L AgNPs had the best results for chlorophyll content, with values of 62.64 and 62.44 SPAD unit, respectively; however, there was no significant difference in the second season. The treated plants with 50% compost + AgNPs 20 mg/L for the first season and 50% compost + AgNPs 30 mg/L for the second season had the greatest percentages of LMEs (8.31% for the first season and 8.34% for the second season).

### 2.6. Antibacterial Activity of A. curassavica Methanolic Leaf Extracts

The leaf methanolic extracts (LMEs) from *A. curassavica* plants treated with various levels of compost (*v*/*v*) + AgNPs (mg/L) are listed in Table 5 for their antibacterial activities. All of the LME concentrations utilized had a noticeable impact on the growth of *Dickeya solani* and *Pectobacterium atrosepticum*. By gradually raising the LME concentration, the inhibition zones (IZs cm) against the development of both bacteria were raised.

The LMEs at the concentrations of 4000, 4000, 4000, and 2000 mg/L, respectively, from the plants treated with compost (*v*/*v*) + AgNPs (mg/L) at the levels of 50% + 30, 25% + 30, 50% + 20, and 50% + 30, respectively, were found to have the highest IZs of 2.43, 2.2, 2.13, and 2.03 cm, reported against the growth of *D. solani*.

The LMEs at the concentration of 4000 mg/L of plants treated with compost (*v*/*v*) + AgNPs (mg/L) at the levels of 50% + 30, 25% + 30, and 50% + 20, respectively, showed the highest IZs of 2.76, 2.73, and 2.46 cm against the growth of *P. atrosepticum*. These values were followed by the IZ values of 2.2, 2.16, and 2.03 cm from the LMEs of plants treated with compost (*v*/*v*) + AgNPs (mg/L) at the levels of 50% + 30, 50% + 20, and 25% + 30, respectively.

Additionally, the impact of the LMEs was significantly affected by the treatments of *A. curassavica* plants with compost + AgNPs at various doses because bacterial growth was inhibited. Plants treated with 25% or 50% compost + AgNPs 30 mg/L showed the greatest antibacterial activity of LMEs.

The determined minimum inhibitory concentrations (MICs) of the LMEs (Table 6) ranged from 15 to 250 mg/L for the development of *D. solani* in comparison to 30 mg/L (control Gentamicin), and from 30 to 250 mg/L in comparison to 35 mg/L (control Gentamicin) for the growth of *P. atrosepticum*. The MIC values against the development of D. solani in the LME of plants treated with amounts of compost (*v*/*v*) + AgNPs (mg/L) of 50% + 30 and 50% + 20, respectively, were 15 and 30 mg/L.

The LME of plants treated with amounts of compost (*v*/*v*) + AgNPs (mg/L) of 25% + 30 and 50% + 30 had the best MIC value against *P. atrosepticum* growth, which was 30 mg/L. The most effective concentration of LME on the growth of bacteria was obtained by treating plants with 25% or 50% compost + AgNPs 30 mg/L.

### 2.7. Phenolic Compounds in Leaf Extracts

**Figure 5:**
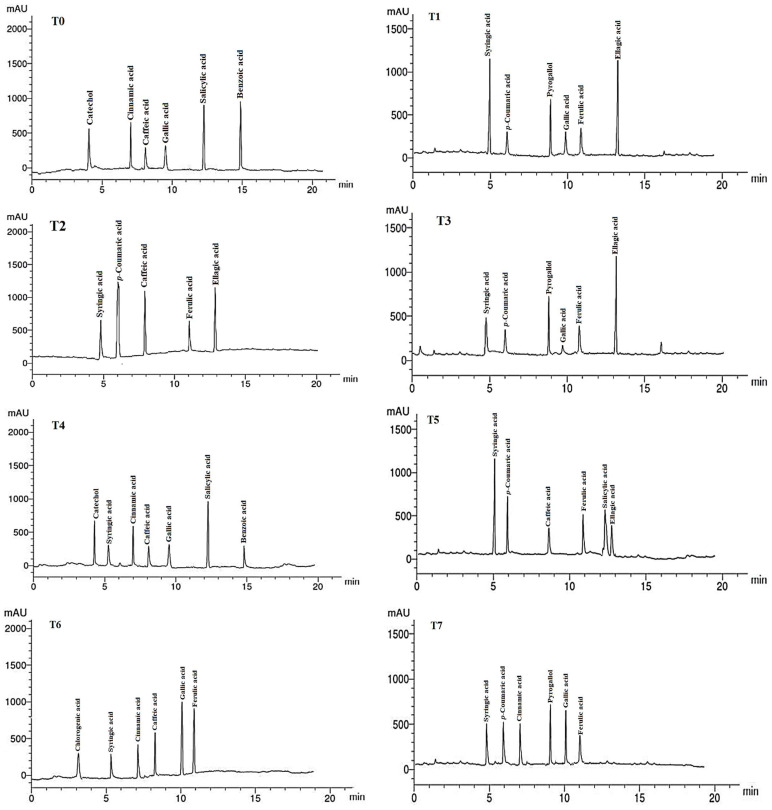

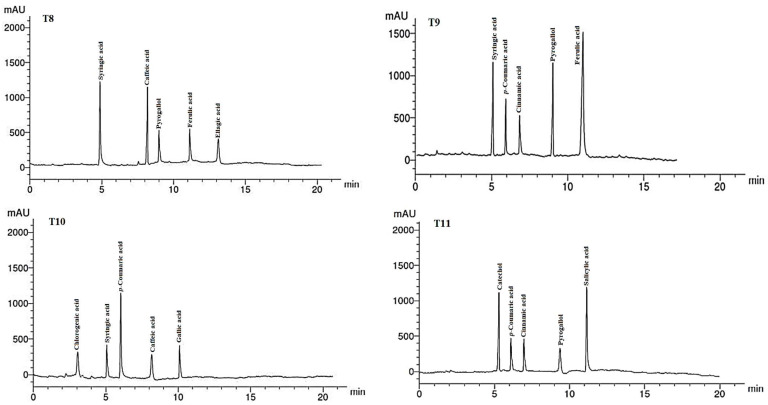
HPLC chromatograms of the identified phenolic compounds in leaf methanol extract from *Asclepias curassavica*. For the legends of T0–T11, see Table 7.

The phenolic compounds (PCs) found in the LMEs of *A. curassavica* by the HPLC analysis are listed in Table 7 and their chromatographic analysis is shown in Figure 5. In the control treatment, benzoic acid (15.08 µg/g), salicylic acid (14.68 µg/g), catechol (8.12 µg/g), and cinnamic acid (6.89 µg/g) were the PCs with the highest abundance in the LME. The primary PCs in the LME from the plants treated with 0% compost + 10 mg/L AgNPs were ellagic acid (12.62 µg/g), syringic acid (11.22 µg/g), and pyrogallol (6.78 µg/g).

The most prevalent PCs in the LME of plants treated with 0% compost + 20 mg/L AgNPs were p-coumaric acid (10.45 µg/g), ellagic acid (8.69 µg/g), and caffeic acid (8.15 µg/g). The two compounds with the highest PC concentrations found in the LME from the plants treated with 0% compost + 30 mg/L AgNPs were ellagic acid (15.39 µg/g) and pyrogallol (9.12 µg/g).

Plants treated with 25% compost + 0 mg/L AgNPs showed the presence of salicylic acid (15.36 µg/g), catechol (8.22 µg/g), and cinnamic acid (7.14 µg/g) as the primary PCs in the LME. Syringic acid (14.32 µg/g) and *p*-coumaric acid (10.45 µg/g) were found to be the main PCs in the LME in the plants treated with 25% compost + 10 mg/L AgNPs, whereas gallic acid (10.33 µg/g) and ferulic acid (9.87 µg/g) were the predominant PCs in plants treated with 25% compost + 20 mg/L AgNPs.

Pyrogallol (10.23 µg/g) and gallic acid (9.44 µg/g) were found to be the primary PCs in plants treated with 25% compost + 30 mg/L AgNPs. In plants treated with 50% compost + 0 mg/L AgNPs, syringic acid (13.22 µg/g) and caffeic acid (12.74 µg/g) were the two PCs that were most prevalent.

The four PCs with the highest concentrations were ferulic acid (16.74 g/g), syringic acid (12.31 g/g), pyrogallol (11.87 g/g), and p-coumaric acid (9.14 g/g) in the LME of plants treated with 50% compost + 10 mg/L AgNPs. The most prevalent PC in LME from the plants treated with 0% compost + 20 mg/L AgNPs was p-coumaric acid (11.42 g/g). Salicylic acid (11.98 g/g) and catechol (8.36 g/g) were present in the LME of the plants treated with 50% compost + 30 mg/L AgNPs as the major PCs.

### 2.8. Flavonoid Compounds in Leaf Extracts

**Figure 6:**
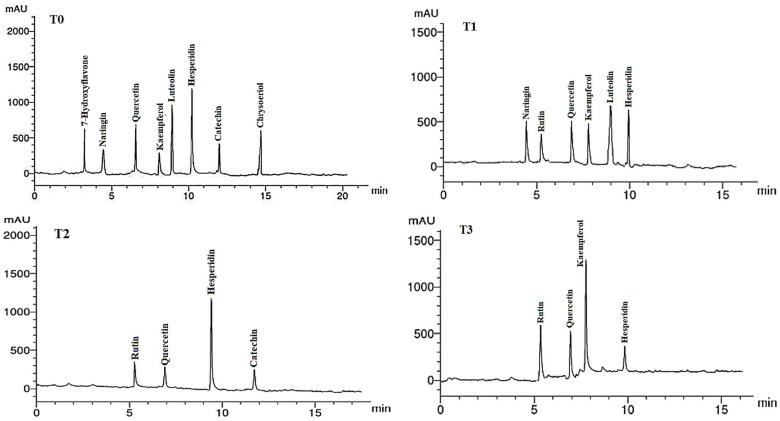

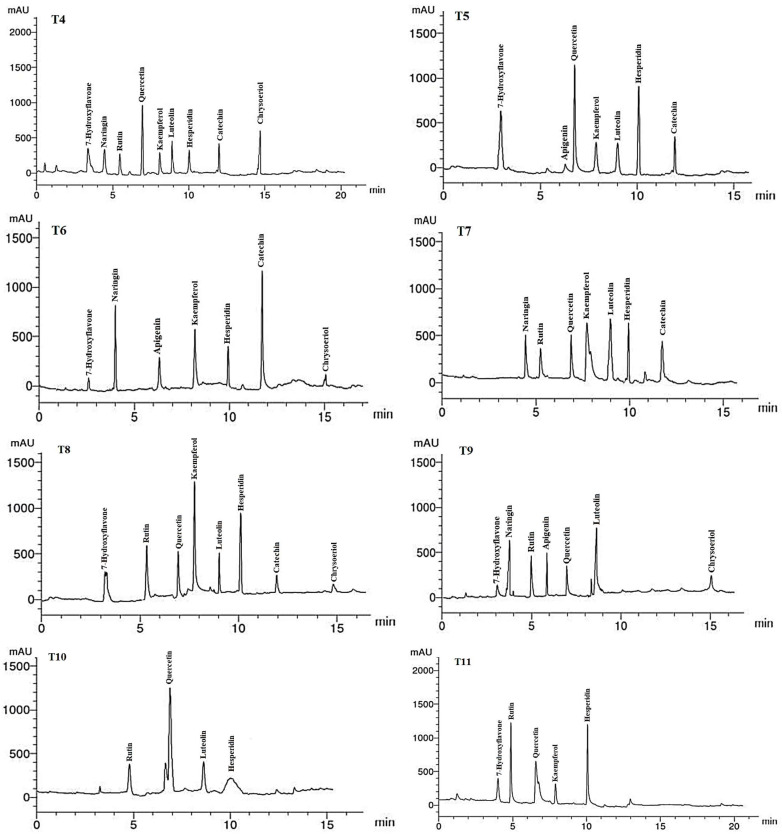
HPLC chromatograms of the identified flavonoid compounds in leaf methanol extracts from *Asclepias curassavica*. For the legends of T0–T11, see Table 7.

The flavonoid compounds (VCs) identified by HPLC analysis in LME of *A. curassavica* are shown in Table 8 and Figure 6. Hesperidin (12.45 µg/g), luteolin (9.58 µg/g), quercetin (8.25 µg/g), and chrysoeriol (7.89 µg/g) were the most prevalent VCs in the LME in the control treatment. The highest concentrations of VCs found in the LME of plants treated with 0% compost + 10 mg/L AgNPs were hesperidin (6.71) and luteolin (6.65 µg/g).

Hesperidin (11.23 µg/g) and rutin (3.02 µg/g) were the two most prevalent VCs found in the LME of plants treated with 0% compost + 20 mg/L AgNPs. The two most prevalent VCs in the LME of plants treated with 0% compost + 30 mg/L AgNPs were kaempferol (9.63 µg/g) and rutin (6.11 µg/g). The primary VCs present in the LME of plants treated with 25% compost + 0 mg/L AgNPs were quercetin (10.22 µg/g) and chrysoeriol (8.63 µg/g).

In plants treated with 25% compost + 10 mg/L AgNPs, the primary VCs in the LME were found to be quercetin (14.23 µg/g), hesperidin (11.26 µg/g), and 7-hydroxyflavone (7.88 µg/g). In the LME of plants treated with 25% compost + 20 mg/L AgNPs, the three largest VCs were catechin (17.58 µg/g), naringin (11.82 µg/g), and kaempferol (8.41 µg/g).

The primary VCs in the LME of plants treated with 25% compost + 30 mg/L AgNPs were kaempferol (7.08 µg/g), luteolin (6.65 µg/g), and catechin (5.00 µg/g). The two compounds with the highest concentrations of VCs in the LME of plants treated with 50% compost + 0 mg/L AgNPs were kaempferol (18.84 µg/g) and hesperidin (12.79 µg/g). The two VCs with the highest concentrations in the LME of plants treated with 50% compost + 10 mg/L AgNPs were luteolin (10.73 µg/g) and naringin (6.84 µg/g). The most abundant VC in the LME of plants treated with 50% compost + 20 mg/L AgNPs was quercetin (14.56 µg/g). Hesperidin (11.06 µg/g), rutin (10.54 µg/g), and quercetin (6.42 µg/g) were the most abundant VCs in the LME of plants treated with 50% compost + 30 mg/L AgNPs.

## 3. Discussion

The *Asclepias curassavica* L. plants (Asclepiadaceae family) or Apocynaceae family were treated with compost and silver nanoparticles (AgNPs), and the results indicated some positive effects on the vegetative growth parameters (plant height, plant diameter, number of branches/plant, leaf area, total fresh weight, total dry weight), total chlorophyll (SPAD unit), and the percentage of leaf methanol extracts (LMEs) in both successive seasons. The LMEs taken from the plants treated with the investigated treatments also contained a number of phenolic and flavonoid chemicals that were identified using HPLC analysis.

The results of the vegetative parameters and all of the photosynthetic pigment contents of fenugreek plants were enhanced by the foliar application of AgNPs concentrations of 20 and 40 mg/L [60]. The considerable promotion of photosynthesis by AgNPs, which was strongly associated with the alteration in nitrogen metabolism [61,62], was noteworthy. The action of AgNPs in suppressing ethylene signaling in the fenugreek plant may be the reason for the induced growth increases brought on by varied AgNPs concentrations [63]. The increased growth characteristics, photosynthetic pigments, and IAA of treated plants may be responsible for these increases in yield and chemical components [60].

Vinblastine from the *Catharanthus roseus* plant (Apocynaceae family) was isolated in high concentrations, which were visible in explants treated with 75 mg/L SNPs and 50 mg/L AgNPs [64]. Different doses of AgNPs had a an impact on the callus proliferation and increased the callus biomass of *Caralluma tuberculata* (family Apocynaceae); using AgNPs at 60 mg/L in combination with 0.5 mg/L2,4-D and 3.0 mg/L BA, the maximum fresh and dry biomass buildup of callus was noted [65]. The average number of leaves, number of branches, and carbohydrate content of the leaves of sunflower plants were all positively impacted by foliar spraying with 50 mL/L of AgNPs. There was no significant interaction among organic fertilizers, AgNPs, and salicylic acid on the vegetative parameters of sunflowers [66]. Different methods of applying AgNPs to the lotus (*Nelumbo nucifera*) increased plant height, leaf diameter, fresh leaf weight, dried leaf weight, and various biochemical characteristics compared to the control. The diameter and chlorophyll content of upright and floating leaves showed a positive correlation with dry leaf mass [67].

AgNPs improved plant qualitative and quantitative yield and increased plant yield and biochemical content of garden thyme (*Thymus vulgaris* L.) exposed to UV-B stress [68].

The growth of the Lilium cv. Mona Lisa was encouraged by soaking its bulbs in various concentrations of AgNPs, as shown by the increased accumulation of leaf and bulb biomass and the hastened flowering. The treated plants also produced more flowers and flowered for a longer period of time. The leaves gained the most chlorophyll a, chlorophyll b, and carotenoids with 100 ppm AgNPs [69]. All concentrations of applied colloidal AgNPs accelerated flowering, increased bulb diameter, and increased the fresh weight of the aboveground portion of the lily plants and bulbs [70]. The quality and quantity of cut flowers and bulbs produced by lily plants increased with the use of AgNPs solutions at various concentrations. All morphological traits rose on average by 26.29% with AgNPs compared to controls, with the exception of vase life [71]. The tulips that were exposed to 100 mg/L AgNPs flowered earlier and had longer stems for cut flowers, larger petals, and greater stem diameter [72].

One of the most commonly utilized nanomaterials is silver nanoparticles (AgNPs); however, studies on plants have shown that AgNPs are hazardous to them at the molecular, cellular, and physiological levels [7,73]. AgNPs are been released have the capacity to penetrate various media and finally reach plant rhizospheres [74,75]. Another result is that the AgNPs are inevitably absorbed by crops and readily enter the food chain [76], having an impact on both food production and food quality as well as posing a risk to human health [77]. When sprayed on rice plants at a concentration of 60 µg/mL, AgNPs not only infiltrated the cell wall but also disrupted the cell shape and structural components and inhibited root growth since AgNPs may only cause harm to plants when utilized in concentrations over a certain point [78]. Additionally, they cause root elongation in *Arabidopsis thaliana* [79] and both vegetative growth and root elongation in *Lolium multiflorum* [80]. AgNPs inhibit plant growth by impairing various stages of cell division and collapsing root cortical cells, epidermis, and root caps [80,81,82].

The absorbed AgNPs altered the structure of the thylakoid membrane due to the AgNPs’ accumulation in leaves, which reduced the amount of chlorophyll and inhibited plant growth [79]. After being exposed to 50 mg/L of AgNPs, *Vigna radiata* seedlings’ total chlorophyll content drastically decreased [83]. As a result of exposure to AgNPs, numerous plants were reported to have significantly less total chlorophyll content [84,85,86]. AgNPs also had an impact on the homeostasis of water and other small molecules within the plant body, as well as the balance between the oxidant and antioxidant systems [79].

Compost has been demonstrated to improve soil structure, increase permeability and infiltration, and increase soil organic matter and water-holding capacity [87,88]. If the compost was tilled into the soil, *Asclepias tuberosa* transplants benefited more frequently from the compost’s better growth and reproduction. Compost had no benefit for the production of flower and seeds. It can be helpful in promoting better growth in prairie plots, but its use can have unfavorable effects in other conditions [89]. Another study revealed that the maximum plant growth of *Acacia nilotica* in pot trials was recorded when a 75% compost level was applied, while the minimum plant growth in the field trial was observed without any compost application. The increase in growth of *A. nilotica* plants was reported with the increases in the amount of compost mixture when a 100% compost level was applied, while the minimum was observed [90].

The combination of biochar–mineral complex and compost stimulated the microbial process in organic farming, leading to better production of vegetables by improving the soil properties [91]. Compost at the rate of 15 ton/feddan enhanced plant height, number of branches, total fresh or dry weight/plant and per feddan, chemical percentages, and chlorophyll a, b, and a + b of dragonhead (*Dracocephalum moldavica*) plants [92]. When the proportion of compost in the media increased, seedlings of *Angelica arch-angelica, Marrubium vulgare*, and *Thymus vulgaris* had higher shoot and root dry weights and mineral concentrations [93].

Under normal or saline–sodic soil conditions, the application of compost exhibited a positive impact on the growth parameters of plants [94]. Compost’s high organic content boosts microbial activity in soils with high salinity, making more nutrients and minerals available to plants, which stimulates crop growth and yield [95,96,97,98].

In the present work, the growth of the two soft rot bacteria *Dickeya solani* and *Pectobacterium atrosepticum* was inhibited by the treatment of methanol leaf extracts from the collected plants with various treatments of compost and AgNPs in the current study. The antimicrobial activity of plant extract is due to the presence of secondary metabolites such as tannin, saponin, phenolics, flavonoids, and glycosides [99,100]. The growth of *Bacillus subtilis, Staphylococcus aureus, Escherichia coli*, and *Klebsiella pneumoniae* were all inhibited by the methanol extract of *A. curassavica*, but *Proteus vulgaris* was not [101]. The methanolic extracts of *A. curassavica* were observed to have antimicrobial activity against the growth of *Bacillus subtilis*, *Staphylococcus aureus*, *Proteus vulgaris*, *Escherichia coli*, and *Klebsiella pneumoniae* at various levels [102].

The *Pinus halepensis* cone extract’s n-butanol fraction, with inhibition zones (IZs) of 14.33 mm and 12.33 mm, respectively, had the highest activity against *D. solani* and *P. atrosepticum* at a concentration of 2000 g/mL [103]. The oily extract from *Bougainvillea spectabilis* was found to be effective against the growth of *D. solani* at a concentration of 4000 g/mL, with an IZ value of 12.33 mm [104]. The growth of *P. atrosepticum* was significantly inhibited by an ethanol extract of *Moringa oleifera* seeds from ripened pods that contained the plant’s primary polyphenolic components vanillic acid, benzoic acid, naringenin, chlorogenic acid, and myricetin [105]. Furthermore, phenolic and flavonoids from plant extracts have demonstrated significant antimicrobial properties. For example, ferulic acid showed antibacterial activity against *D. solani*, with an IZ ranging from 6.00 to 25.75 mm at various doses, but chlorogenic acid was ineffective [104].

The levels of compost and AgNPs treatments had an impact on the contents of numerous phenolic and flavonoid components in the MLEs from *A. curassavica* plants. When compared to untreated plants, AgNPs treatments resulted in large increases in the total phenolic, flavonoid, and tannin contents [60]. *A. curassavica*, with its bioactive compounds (calactin, calotropin, calotropagenin, coroglaucigenin, asclepin, asclepain CI, asclepain CII, asclepine (asclepiadin), uscharidin), was used traditionally in different populations [106]. Sixteen flavonoids, all of which are derivatives of the flavonols quercetin and kaempferol, were isolated from the leaf material of *Asclepias* [107]. The flavonoids glyeosides-quercetin 3-O-(2″,6″-α-L-dirhamnopyranosyl)-β-D-galactopyranoside, quercetin 3-O-β-dglucopyranosyl-(1→6)-β-D-galactopyranoside, quercetin 3-O-(2″-O-~t-L-rhamnopyranosyl)-β-D-galactopyranoside, quercetin 3-O-α-L-rhamnopyranosyl-(1→6)-β-D-glucopyranoside, quercetin 3-O-β-D-galactopyranoside, quercetin 3-O-β-D-glucopyranoside, and quercetin 3-O-(2″,6″-α-L-dirhamnopyranosyl)-β-D-glucopyranoside were isolated and characterized from this plant [108]. The flavonoids quercetin 3-O-D-glucopyranosyl (1→6)-β-D-galactopyranoside, quercetin 3-0-(2″,6″-α-L-dirharnnopyranosyl)-β-D-galactopyranoside, and rutin were isolated from *A. curassavica* ethanolic leaf extract [109].

Flavonoids, fixed oils, phenols, quinines, tannin, terpenoid, glycosides, coumarins, sugars, xanthoprotein, saponin, and steroids were present in the leaf, stem, and root extracts of *A. curassavica* [102,110,111]. Steroids, glycosides, phenols, and saponins were found in the methanol extracts of *A. curassavica* root and leaf, while flavonoids and resins were present in smaller amounts [102]. The total phenolics in *A. muricata* treated with various concentrations of AgNPs (250–1000 ppm) did not differ significantly. *A. muricata* leaves treated with AgNPs differed significantly from the control in terms of their flavonoid content [112].

To the best of our knowledge, this is the first study to examine how different compost and AgNPs combination treatments affect the productivity, growth, and phytochemicals of *A. curassavica* plants as measured over the course of two successive growing seasons. Although using synthesized nanoparticles as biostimulants has many intrinsic advantages, problems such as toxicity at high concentrations and dangerous disposal to the environment may limit their continuing usage, which opens new research opportunities.

A further limitation is that the stability of nanoparticles in the environment is another tough feature in utilizing them. The size of the particles and their affinity for other environmental components heavily influence the suspension’s stability. The poorer stability of the metal nanoparticles in nature makes them more susceptible to oxidation in the air. These nanoparticles are kept in a specialized environment of inert gases because they cannot be kept in regular environmental conditions for use in the future [113,114].

## 4. Materials and Methods

### 4.1. Synthesis of Silver Nanoparticles (AgNPs)

Silver nanoparticles (AgNPs) were synthesized chemically using analytical-grade ethanol, silver nitrate (AgNO_3_), and sodium borohydride (NaBH_4_). In this procedure, sodium borohydride served as a reducing agent while ethanol served as a stabilizing agent. A homogeneous solution was created by dissolving 500 mg of AgNO_3_ in 20 mL of ethanol and stirring it with a magnet stirrer for 1 h [54]. This step is necessary for the production of silver particles. After that, 500 mg of NaBH_4_ was added to this solution, one drop at a time. When the solution changes from clear to black, this means that AgNPs are being produced. The precipitation was then collected, filtered, and repeatedly washed with ethanol and deionized water. The dried nanoparticles were maintained in a dark bottle for further characterization studies.

### 4.2. Characterization of AgNPs

The morphology, surface, and shape of the AgNPs were characterized by scanning electron microscopy (SEM) at 10 kV (JSM-6360 LA, JEOL, Tokyo, Japan) with a 3 mm working distance and transmission electron microscopy (TEM) using the JEM-2100 microscope (JEOL, Tokyo, Japan). The presence of the produced AgNPs was investigated using a UV-visible spectrophotometer (Shimadzu, Tokyo, Japan), with the reduction of pure Ag+ ions verified via measurement at UV-245 double-beam (200–1000 nm). The X-ray diffraction analysis (XRD) patterns were registered in a diffractometer (Shimadzu XRD-6100) using CuKα radiation (k = 1:5406 A°) operated at a voltage of 40 kV and a current of 30 mA. Data were collected over a 2θ range of 5–80°, 0.0200 steps, and 10 s of counting time per step. Based on the XRD peak widths, the crystallite domain size was determined. They must be free of non-uniform moieties, according to the assumption. With the help of the Debye–Scherrer equation, the average size of the AgNPs may be determined. A particle size analyzer (PSA, MALVERN, ZETASIZER Ver.6.20) was used to examine particle size distribution. Quartz was used to examine the material, Thetes, the temperature was set at 25 °C, and pure water was utilized for viscosity and refractive index data, resulting in high size resolution. Furthermore, an assessment of the surface functional groups of the AgNPs that were synthesized was conducted via Fourier transform infrared spectroscopy (FTIR) utilizing the KBr-disc method within the 400–4000 cm^−1^ range.

### 4.3. Experimental Field Design and Data Recorded

The field study on *Asclepias curassavica* plants was conducted over the course of two successive growing seasons, 2020 and 2021, at the Nursery of Department of Floriculture, Ornamental Horticulture and Garden Design, Faculty of Agriculture, Alexandria University, Egypt. The plant with its voucher number Z0001 was identified by Dr. Hany M. El-Naggar (Department of Floriculture, Ornamental Horticulture and Garden Design, Faculty of Agriculture (El-Shatby), Alexandria University, Alexandria).

Clay and sand (2:1 *v*/*v*) were used as the growing media for *A. curassavica* plants, and two amounts of compost were applied when the soil was prepared for the two seasons, as shown in Table 9. AgNPs were sprayed on the plants three times: on the first of May, the first of June, and the first of July for the two seasons. The concentrations used were 0, 10, 20, and 30 mg/L. Table 10 displays the results of the compost’s chemical analysis.

In both seasons, measurements were taken of the plant’s height (cm), diameter (cm), number of branches/plant, leaf area, total fresh weight (g), total dry weight (g), total chlorophyll (SPAD unit), and the percentage of extracts.

The extraction yield of *A. curassavica* plants was calculated using the following equation: extraction yield (%) = W1/W2 × 100, where W1 is the mass of leaf crude extract and W2 is the mass of the leaf sample [115].

### 4.4. Preparation of Plant Methanol Extracts

*A. curassavica* leaves were air dried at room temperature and transferred to powder using a grinder. About 50 g from the powdered leaves was extracted by the methanol solvent (150 mL) for 72 h at room temperature. The solvent was removed and the extracts were concentrated and collected in separate bottles and stored at 4 °C until further analysis [116].

### 4.5. Antibacterial Activity

The selected phytopathogenic bacteria, *Dickeya solani* (LT592258) and *Pectobacterium atrosepticum* (LN851554), were obtained from the Department of Plant Pathology, Faculty of Agriculture (El-Shatby), Alexandria University (Alexandria, Egypt). Methanolic extract of *A. curassavica* was dissolved in 10% dimethyl sulfoxide (DMSO) and prepared at different concentrations (4000, 2000, 1000, and 500 μg/mL).

Antibacterial activity was determined using the disc diffusion method [117,118], where the autoclaved filter paper discs 5 mm in diameter were used and each disc received 40 μL of the prepared concentrations (4000, 2000, 1000, and 500 μg/mL). The minimum inhibitory concentrations (MICs) were determined by serial dilution of the extracts ranging from 15 to 4000 μg/mL [119]. Negative (10% DMSO) and positive (gentamicin 20 μg/disc) controls were used, and all tests were performed in triplicate.

### 4.6. HPLC Analysis of Phenolic and Flavonoid Components

The phenolic components from the methanol extracts of *A. curassavica* leaves were categorized by HPLC (Agilent 1100, USA). A binary LC pump, a UV/Vis detector, and a C18 column (125 mm, 4.60 mm, 5 µm) make up this apparatus. The Agilent ChemStation was used to acquire and analyze chromatograms. A gradient mobile phase of two solvents—Solvent A (MeOH) and Solvent B [Acetic acid in H_2_O (1:25)]—was used to separate phenolic acids. The gradient program began with 100% B and remained there for 3 min. This was followed by 5 min of 50% eluent A, 2 min of 80% eluent A, 5 min of 50% eluent A, 2 min of 80% eluent A, 5 min of 50% eluent A, 5 min of 50% eluent A, and 5 min of detection wavelength at 250 nm. As a result, the phenolic components were arranged in order to authenticate standard components by this mobile phase [120,121]. For the flavonoid compounds, HPLC (Agilent 1100), composed of two LC pumps, a UV/Vis detector, and C18 column (250 mm × 4.6 mm, 5 µm), was used. The mobile phase was acetonitrile (A) and 0.2% (*v*/*v*) aqueous formic acid (B) with an isocratic elution (70:30) program. The detection wavelength was set at 360 nm [121].

### 4.7. Statistical Analysis

The experiment was statically analyzed using CoStat program ver., 6.303 (CoHort software, Monterey, CA, USA). A completely randomized design [122] was performed and the means were equated by the Least Significant Difference (LSD) at 0.05 level of probability [123]. The data were expressed as means ± 2SD values and were deemed statistically significant when *p* ≤ 0.05.

## 5. Conclusions

The findings of the current study show that the interaction of compost and AgNPs applications had a positive effect on the vegetative parameters of *A. curassavica* plants, including plant height (cm), plant diameter (cm), branch number/plant, leaf fresh weight (g), leaf dry weight (g), and leaf area (cm^2^), gradually increasing with concentration compared to untreated plants. The chemical constituents of *A. curassavica* plants, such as their chlorophyll content, leaf methanol extract percentages, phenolic compounds, and flavonoid compounds, were also impacted by the treatments. Generally, the best outcomes were achieved with for the majority of the assessed parameters. We recommend that the plants treated with 50% compost containing 20 or 30 mg/L AgNPs be employed in field experiments at a wide scale and in subsequent research.

## Figures and Tables

**Table 1:** The X-ray parameters of AgNPs.

Diffraction Angles (2θ) Degree	I/I_o_	d (Å)	(h k l)	Type	Structure	Full Width at Half Maximum (FWHM)	D (nm)
37.51°	100	2.449	(111)	Ag	Fcc*	0.24020°	0.3911
42.81°	52	2.03	(200)	Ag	fcc	0.34080°	21.777
66.44°	39	1.488	(220)	Ag	fcc	0.32722°	28.910
79.29°	17	1.255	(311)	Ag	fcc	0.29033°	27.230

* fcc: face-centered cubic material.

**Table 2:** XRD three characteristic peaks calculated, d-spacing, and the expected 2θ positions.

2θ Measured	d-Spacing Corresponding Value	Expected 2θ Positions
37.51°	0.2355	36.35°
42.81°	0.1972	41.44°
66.44°	0.1412	62.70°
79.29°	0.1205	72.72°

**Table 3:** Vegetative parameters of *A. curassavica* plants as affected by the treatments of compost and AgNPs in two seasons.

Treatment		Height (cm)		Diameter (cm)		No. Branches/Plant		Leaf Area (cm^2^)		Total F.W. (g)		Total D.W. (g)	
Compost *v*/*v*	AgNPs mg/L	1st (ns)	2nd (ns)	1st	2nd (ns)	1st (ns)	2nd	1st (ns)	2nd	1st	2nd	1st (ns)	2nd
0	0	37.83 ± 2.08	43 ± 3.46	37.16 ± 3.05 *^f^*	38.83 ± 3.05	3.36 ± 0.41	3.63 ± 0.70 *^g^*	7.26 ± 1.11	8.45 ± 0.40 *^h^*	37.37 ± 2.59*^f^*	39.20 ± 1.89 *^f^*	12.27 ± 1.58	13.31 ± 1.10 *^fg^*
	10	36.61 ± 2.79	41.89± 2.41	37.75 ± 2.17 *^def^*	39 ± 2.64	4.43 ± 1.02	4.43 ± 1.02 *^g^*	8.63 ± 2.10	9.35 ± 1.01 *^gh^*	40.21 ± 2.75 *^f^*	39.33 ± 1.42 *^f^*	12.98 ± 1.90	12.63 ± 1.30 *^g^*
	20	39.66 ± 2.08	45.16 ± 1.45	39.91 ± 0.28 *^cd^*	42 ± 3.60	5 ± 1.2	6.21 ± 2.0 *^f^*	10.32 ± 2.76	9.69 ± 1.74 *^fgh^*	44.59 ± 2.19 *^e^*	44.33 ± 3.54 *^e^*	14.04 ± 0.77	14.01 ± 1.11 *^fg^*
	30	42.72 ± 4.98	46.55 ± 2.14	42.58 ± 2.92 *^ab^*	41.33± 3.21	6.3 ± 0.6	8.2 ± 1.70 *^bcd^*	11.02 ± 2.10	10.54 ± 1.46 *^efg^*	46.83 ± 2.6 *^de^*	48.2 ± 4.50 *^d^*	15.68 ± 1.42	14.53 ± 1.51 *^ef^*
25%	0	48.33 ± 5.85	49.66 ± 6.35	37.58 ± 1.25 *^ef^*	42.92 ± 3.81	6.33 ± 2.51	7.66 ± 2.88 *^cde^*	9.21 ± 2.26	11.09 ± 1.74 *^def^*	50.71 ± 1.72 *^cd^*	50.16 ± 1.95 *^cd^*	16.79 ± 0.66	15.84 ± 3.13 *^df^*
	10	48.16 ± 5.50	51.33 ± 12.89	36.66 ± 3.81 *^f^*	40.83 ± 2.88	6.83 ± 3.51	6.66 ± 1.15 *^ef^*	13.51 ± 1.77	13.42 ± 0.98 *^b^*	53.043 ± 9.92 *^c^*	54.99 ± 2.34 *^b^*	16.51 ± 2.52	17.31 ± 1.081 *^cd^*
	20	55 ± 7.93	61.66 ± 13.77	40.83 ± 3.81 *^bc^*	44.61 ± 7.89	6.55 ± 2.14	8.55 ± 1.53 *^abc^*	15.12 ± 2.14	13.36 ± 2.05 *^bc^*	52.189 ± 3.74 *^c^*	56.55 ± 3.03 *^b^*	17.38 ± 1.32	18.90 ± 0.47 *^bc^*
	30	52 ± 2	60.44 ± 7.72	40 ± 1 *^cd^*	44.61 ± 2.27	9.44 ± 1.01	9.22 ± 0.77 *^ab^*	16.21 ± 2.45	16.21 ± 3.38 *^a^*	58.45 ± 8.44 *^b^*	61.63 ± 3.32 *^a^*	19.21 ± 1.52	20.03 ± 0.38 *^b^*
50%	0	48.55 ± 3.35	58.16 ± 3.51	39.776 ± 1.83 *^cde^*	42.44 ± 50	6.78 ± 0.38	7.05 ± 2.83 *^def^*	10.59 ± 0.30	11.92 ± 1.78 *^cde^*	50.09 ± 7.34 *^cd^*	51.41 ± 1.76 *^c^*	15.90 ± 1.36	19.03 ± 1.11 *^b^*
	10	54.16 ± 4.72	55.16 ± 6.02	40.41 ± 1.44 *^bc^*	42.92 ± 6.29	5.55 ± 1.01	6.33 ± 2.30 *^f^*	11.44 ± 2.28	12.22 ± 1.25 *^bcd^*	59.97 ± 7.56 *^ab^*	55.31 ± 2.89 *^b^*	17.79 ± 2.08	19.09 ± 1.15 *^b^*
	20	58.05 ± 18.58	59.44 ± 2.34	43.69 ± 4.79 *^a^*	42.36 ± 4.10	9.16 ± 4.16	9.66 ± 2.08 *^a^*	13.03 ± 0.96	13.61 ± 1.61 *^b^*	62.73 ± 5.88 *^a^*	62.82 ± 4.53 *^a^*	19.43 ± 1.44	22.91 ± 2.57 *^a^*
	30	53.55 ± 1.38	59.77 ± 13.08	40.52 ± 1.29 *^bc^*	45 ± 5.56	8.83 ± 2.08	9 ± 1 *^ab^*	14.40 ± 2.81	13.66 ± 1.19 *^b^*	59.33 ± 5.67 *^ab^*	63.67 ± 5.70 *^a^*	19.51 ± 2.13	24.10 ± 2.69 *^a^*

Values are means ± 2SD. The means with the same letter/s within the same column have no significant difference according to LSD0.05. ns: Not significant.

**Table 4:** Effect of compost and AgNP treatments on the biochemical components of *A. curassavica* plants throughout two seasons.

Treatment		Chlorophyll (SPAD Unit)		Extract Yield (%)	
Compost (*v*/*v*)	AgNPs (mg/L)	1st (ns)	2nd	1st	2nd (ns)
0	0	41.1 ± 2.02	44.04 ± 3.18 *^e^*	5.55 ± 0.56 *^cdef^*	4.82 ± 0.92
0	10	40.68 ± 3.16	43.76 ± 1.83 *^e^*	4.84 ± 1.01 *^ef^*	5.15 ± 1.39
0	20	45.40 ± 3.25	45.72 ± 3.69 *^de^*	5.80 ± 0.58 *^cde^*	5.25 ± 0.70
0	30	48.96 ± 1.80	47.05 ± 1.17 *^de^*	6.62 ± 0.64 *^bc^*	5.92 ± 0.25
25%	0	48.24 ± 2.97	46.37 ± 7 *^de^*	5.45 ± 1.35 *^def^*	5.13 ± 1.62
25%	10	54.92 ± 6.28	53.03 ± 12.08 *^bc^*	5.94 ± 1.67 *^cde^*	5.87 ± 0.69
25%	20	61.83 ± 6.65	60.89 ± 15.03 *^a^*	6.50 ± 1.97 *^bcd^*	6.58 ± 1.79
25%	30	62.64± 8.01	58.81 ± 7.09 *^a^*	8.03 ± 2.18 *^a^*	6.96 ± 1.05
50%	0	43.73 ± 8.04	46.44 ± 7.09 *^de^*	4.55 ± 1.39 *^f^*	5.75 ± 0.78
50%	10	49.50 ± 9.09	48.55 ± 13.41 *^cd^*	7.25 ± 2.03 *^ab^*	6.11 ± 1.83
50%	20	55.09 ± 6.86	57.02 ± 6.21 *^ab^*	8.31 ± 1.16 *^a^*	7.93 ± 0.70
50%	30	62.44 ± 8.81	53.70 ± 9.37 *^b^*	7.96 ± 0.37 *^a^*	8.34 ± 0.99

Values are means ± 2SD. This indicates that, according to LSD0.05, there are no significant differences for the same letter or letters inside the same column. ns: At a 0.05 level of probability, not significant. ns: Not significant.

**Table 5:** Inhibition zones of the methanol extracts from plants treated with compost (*v*/*v*) + AgNPs (mg/L) treatments.

Treatment	Inhibition Zones against *Dickeya solani* Growth (cm)				
	Methanol Extract Concentration (mg/L)				
Compost (*v*/*v*) + AgNPs (mg/L)	Control	4000	2000	1000	500
0+ 0	0	1.23 ± 0.11	1.16 ± 0.11	1.06 ± 0.11	0.9 ± 0
0 + 10	0	1.16 ± 0.11	1.06 ± 0.30	1.03 ± 0.30	0.86 ± 0.11
0 + 20	0	1.26 ± 0.11	1.2 ± 0	1.13 ± 0.11	0.96 ± 0.23
0 + 30	0	1.43 ± 0.11	1.2 ± 0.2	1 ± 0.2	0.8 ± 0
25% ± 0	0	1.1 ± 0	1.1 ± 0	1 ± 0	0.93 ± 0.81
25% ± 10	0	1.2 ± 0	1.133 ± 0.11	1.133 ± 0.11	1.1 ± 0
25% ± 20	0	1.53 ± 0.11	1.33 ± 0.11	1.2 ± 0	1.1 ± 0.2
25% ± 30	0	2.2 ± 0.2	1.8 ± 0.2	1.33 ± 0.11	1.2 ± 0.2
50% ± 0	0	1.3 ± 0	1.2 ± 0	1.2 ± 0	1.2 ± 0
50% ± 10	0	1.56 ± 0.11	1.43 ± 0.11	1.3 ± 0	1.16 ± 0.11
50% ± 20	0	2.13 ± 0.23	1.8 ± 0.2	1.5 ± 0	1.3 ± 0.2
50% ± 30	0	2.43 ± 0.11	2.03 ± 0.11	1.63 ± 0.30	1.36 ± 0.11
Gentamicin 20 mg/disc	3				
LSD 0.05	0.103				
Treatment	Inhibition zones against *Pectobacterium atrosepticum* growth (cm)				
0	0	1.6 ± 0.2	1.56 ± 0.11	1.23 ± 0.11	0.93 ± 0.11
0 + 10	0	1.26 ± 0.11	1.16 ± 0.11	1.1 ± 0.2	0.9 ± 0
0 + 20	0	1.36 ± 0.23	1.46 ± 0.30	1.26 ± 0.41	0.83 ± 0.11
0 + 30	0	1.63 ± 0.30	1.53 ± 0.11	1.16 ± 0.11	0.8 ± 0
25% ± 0	0	1.46 ± 0.11	1.26 ± 0.11	1.13 ± 0.11	1 ± 0
25% ± 10	0	1.66 ± 0.11	1.36 ± 0.11	1.2 ± 0	1.13 ± 0.11
25% ± 20	0	1.86 ± 0.11	1.63 ± 0.11	1.46 ± 0.11	1.36 ± 0.11
25% ± 30	0	2.73 ± 0.11	2.03 ± 0.11	1.7 ± 0.4	1.3 ± 0.4
50% ± 0	0	1.4 ± 0.2	1.3 ± 0	1.3 ± 0.2	1.2 ± 0.2
50% ± 10	0	1.76 ± 0.30	1.63 ± 0.30	1.46 ± 0.11	1.2 ± 0.2
50% ± 20	0	2.46 ± 0.11	2.16 ± 0.30	1.7 ± 0.2	1.36 ± 0.11
50% ± 30	0	2.76 ± 0.11	2.2 ± 0.2	1.96 ± 0.11	1.43 ± 0.11
Gentamicin 20 mg/disc	3.5				
LSD 0.05	0.137				

**Table 6:** The MIC (mg/L) measured against the growth of *D. solani* and *P. atrosepticum*.

Treatments	MIC (mg/L)	
Compost (*v*/*v*) + AgNPs (mg/L)	*D. solani*	*P. atrosepticum*
0 + 0	250	500
0 + 10	250	250
0 + 20	250	500
0 + 30	500	500
25% + 0	250	250
25% + 10	125	125
25% + 20	60	125
25% + 30	60	30
50% + 0	250	250
50% + 10	125	125
50% + 20	30	60
50% + 30	15	30
Gentamicin 20 mg/disc	30	35

**Table 7:** Phenolic compounds identified in the leaf methanol extracts from *Asclepias curassavica* by HPLC.

Compound	Concentration (µg/g of Methanol Leaf Extract)											
	T0	T1	T2	T3	T4	T5	T6	T7	T8	T9	T10	T11
Chlorogenic acid	ND	ND	ND	ND	ND	ND	3.01	ND	ND	ND	2.03	ND
Catechol	8.12	ND	ND	ND	8.22	ND	ND	ND	ND	ND	ND	8.36
Syringic acid	ND	11.22	4.58	5.22	3.56	14.32	2.10	6.13	13.22	12.31	3.56	ND
p-Coumaric acid	ND	2.36	10.45	3.05	ND	10.45	ND	6.77	ND	9.14	11.42	4.22
Cinnamic acid	6.89	ND	ND	ND	7.14	ND	3.23	5.98	ND	7.24	ND	4.08
Caffeic acid	2.76	ND	8.15	ND	2.78	2.38	4.24	ND	12.74	ND	1.89	ND
Pyrogallol	ND	6.78	ND	9.12	ND	ND	ND	10.23	4.12	11.87	ND	2.49
Gallic acid	3.10	2.14	ND	0.75	3.11	ND	10.33	9.44	ND	ND	3.15	ND
Ferulic acid	ND	3.04	3.66	4.31	ND	6.32	9.87	3.12	5.32	16.74	ND	ND
Salicylic acid	14.68	ND	ND	ND	15.36	6.52	ND	ND	ND	ND	ND	11.98
Ellagic acid	ND	12.62	8.69	15.39	ND	5.13	ND	ND	3.10	ND	ND	ND
Benzoic acid	15.08	ND	ND	ND	2.41	ND	ND	ND	ND	ND	ND	ND

ND: not detected; T0: 100% nursery soil; T1: 0% Compost + 10 mg/L AgNPs; T2: 0% Compost + 20 mg/L AgNPs; T3: 0% Compost + 30 mg/L AgNPs; T4: 25% Compost + 0 mg/L AgNPs; T5: 25% Compost + 10 mg/L AgNPs; T6: 25% Compost + 20 mg/L AgNPs; T7: 25% Compost + 30 mg/L AgNPs; T8: 50% Compost + 0 mg/L AgNPs; T9: 50% Compost + 10 mg/L AgNPs; T10: 50% Compost + 20 mg/L AgNPs; T11: 50% Compost + 30 mg/L AgNPs.

**Table 8:** Flavonoid compounds identified in the leaf methanol extracts from *Asclepias curassavica* by HPLC.

Compound	Concentration (µg/g of Methanol Leaf Extract)											
	T0	T1	T2	T3	T4	T5	T6	T7	T8	T9	T10	T11
7-Hydroxyflavone	7.76	ND	ND	ND	3.55	7.88	0.35	ND	3.11	0.23	ND	2.33
Naringin	2.12	4.01	ND	ND	2.14	ND	11.82	3.52	ND	6.84	ND	ND
Rutin	ND	2.11	3.02	6.11	1.89	ND	ND	2.49	5.47	3.40	5.16	10.54
Apigenin	ND	ND	ND	ND	ND	0.56	3.60	ND	ND	2.63	ND	ND
Quercetin	8.25	4.40	1.98	5.10	10.22	14.23	ND	4.77	4.63	1.55	14.56	6.42
Kaempferol	1.65	3.89	ND	9.63	1.46	4.27	8.41	7.08	18.84	ND	ND	1.44
Luteolin	9.58	6.65	ND	ND	4.06	3.99	ND	6.65	1.45	10.73	4.83	ND
Hesperidin	12.45	6.71	11.23	1.07	2.33	11.26	4.73	4.31	12.79	ND	5.23	11.06
Catechin	3.18	ND	1.53	ND	3.07	5.74	17.58	5.00	1.22	ND	ND	ND
Chrysoeriol	7.89	ND	ND	ND	8.63	ND	0.28	ND	0.87	0.95	ND	ND

ND: not detected; for the legends of T0–T11, see Table 7.

**Table 9:** The treatments of compost and AgNPs used in the present study.

Treatments	Compost (% from the Soil Mixer)	Silver Nanoparticles (AgNPs) (mg/L)
T0	0	0
T1		10
T2		20
T3		30
T4	(25% compost) 25% compost and 75% of nursery soil	0
T5		10
T6		20
T7		30
T8	(50% compost) 50% compost and 50% nursery soil	0
T9		10
T10		20
T11		30

**Table 10:** Chemical analysis of the compost.

Element	Value
Organic carbon (OC)	16.56%
Organic matter (OM)	28.55%
Nitrogen (N)	1.57%
Carbon/Nitrogen (C/N ratio)	10.54
Phosphorus (P)	0.45%
Potassium (K)	2.45%

## Data Availability

Not applicable.
